# Increased Entropy Predicts Adverse Cardiac Events in Patients with High Cardiovascular Risk and Hypertension: A Novel Imaging Parameter Derived from Late Gadolinium Enhancement

**DOI:** 10.31083/RCM26499

**Published:** 2025-05-27

**Authors:** Yunbo Zhang, Lujing Wang, Jin Wang, Xinxiang Zhao

**Affiliations:** ^1^Department of Education, Second Affiliated Hospital of Kunming Medical University, 650000 Kunming, Yunnan, China; ^2^Department of Radiology, Second Affiliated Hospital of Kunming Medical University, 650000 Kunming, Yunnan, China; ^3^Department of Radiology, Yanan Hospital of Kunming City, 650000 Kunming, Yunnan, China

**Keywords:** hypertension, cardiac magnetic resonance, prognosis

## Abstract

**Background:**

Entropy derived from late gadolinium enhancement (LGE) has been shown to correlate with major adverse cardiac events (MACEs) in various cardiac diseases. However, the association between myocardial entropy and MACEs in patients with hypertension (HTN) has not been reported.

**Methods:**

This study recruited 190 patients with high cardiovascular risk and essential HTN who underwent cardiac magnetic resonance (CMR) examination in our hospital between January 2020 and June 2024. HTN patients were followed up for MACEs, which were defined as hospitalization for the occurrence of heart failure, acute coronary syndromes, stroke, or all-cause death. Patients were divided into MACE and non-MACE groups. Cardiac morphology, function, and tissue characteristics were assessed using CMR, and left ventricular (LV) entropy was acquired from LGE images.

**Results:**

Of the 190 patients with HTN, 54 (28.4%) experienced a MACE over a median follow-up period of 12.0 (8.0–27.0) months. LV entropy was significantly higher in patients with MACEs than those without (5.75 ± 0.89 vs. 5.12 ± 1.26; *p* < 0.001). Furthermore, LV entropy was an independent predictor of MACE, even after adjustment for clinical risk factors (odds ratio: 1.569 (1.039–2.369); *p* = 0.032). Receiver operating characteristic curve (ROC) analysis showed the predictive value of LV entropy, with an area under the curve (AUC) of 0.663. Adding LV entropy to the clinical model resulted in a relatively higher AUC (0.813 vs. 0.806) for the prediction of MACEs; however, this was not significantly different from the clinical model alone (*p* = 0.570).

**Conclusions:**

HTN patients with MACEs presented higher LV entropy than patients without MACEs. Furthermore, as an independent predictor of MACEs, LV entropy may help the risk stratification of HTN patients with high cardiovascular risk.

**Clinical Trial Registration:**

ChiCTR2100049160, https://www.chictr.org.cn/showproj.html?proj=130381.

## 1. Introduction

Cardiovascular disease is one of the leading causes of death globally [1]. Recent research has revealed that more than 69% of patients with hypertension (HTN) are at high cardiovascular risk [2]. A previous study found that HTN presents with significant heterogeneity, with different biochemical and hemodynamic mechanisms for increased blood pressure [3]. Left ventricular hypertrophy (LVH) is currently the most commonly used parameter for predicting the outcome of patients with HTN. However, LVH is not detected in all HTN patients and may be only partly responsible for poor prognosis. Furthermore, major adverse cardiac events (MACEs) in HTN patients are known to be associated with myocardial fibrosis, which leads to ventricular dysfunction, myocardial remodeling, and ventricular stiffness [4, 5].

Cardiac magnetic resonance (CMR) examination for late gadolinium enhancement (LGE) is a non-invasive test commonly used to assess myocardial fibrosis. However, a previous study found that HTN patients without LGE still develop MACEs [6], suggesting that LGE may not be an ideal parameter for the detection of all types of myocardial fibrosis. Hence, the accurate risk stratification of patients with HTN using novel tissue parameters remains an important issue for clinicians.

Entropy derived from LGE is a new CMR technique that can provide whole myocardial pixel signal intensity through software post-processing, while also revealing the heterogeneity of the entire myocardial tissue [7]. Myocardium with zero entropy shows perfectly homogeneous pixels, whereas heterogeneous myocardium has higher entropy. In sharp contrast to LGE, entropy allows the assessment of myocardial heterogeneity without significant signal intensity thresholds. Notably, entropy has been found to correlate with prognosis in patients with a variety of heart diseases. For example, entropy can predict the development of malignant arrhythmias in cardiac patients, as well as adverse events in patients with myocardial infarction [8, 9]. To our knowledge, however, there have been no reports on the association between entropy and MACE in patients with high cardiovascular risk and HTN.

Since the presence of myocardial fibrosis in patients with HTN is complex [10], we hypothesized that left ventricular (LV) entropy could be an ideal parameter to assess myocardial heterogeneity in these patients. Therefore, the aim of this research was to study the relationship between entropy and the extent of myocardial heterogeneity in HTN patients, and to explore the predictive value of entropy for MACEs.

## 2. Materials and Methods

### 2.1 Study Population and Clinical Outcomes

This study retrospectively analyzed 190 patients who attended our hospital from January 2020 to June 2024 with a diagnosis of HTN and high cardiovascular risk. The study was approved by the research ethics committee (PJ-202330) of our hospital. All participants signed an informed consent form, and the study complied with the Declaration of Helsinki (clinical registry number: ChiCTR2100049160, https://www.chictr.org.cn/showproj.html?proj=130381). The study population comprised patients with essential HTN who were at high cardiovascular risk, with the aim of investigating early cardiac functional and structural alterations associated with MACEs. Although the clinical registry utilized in this study is labelled “nonischemic cardiomyopathy (NICM),” this designation does not indicate that all enrolled participants exhibited end-stage NICM phenotypes, such as left ventricular dilatation or clinical heart failure. Instead, the study focused on HTN patients as representing an early pathological stage within the NICM spectrum. Accordingly, the enrolled individuals with HTN may have reflected a range of transitional stages in the development and progression of NICM.

**Fig. 1.**
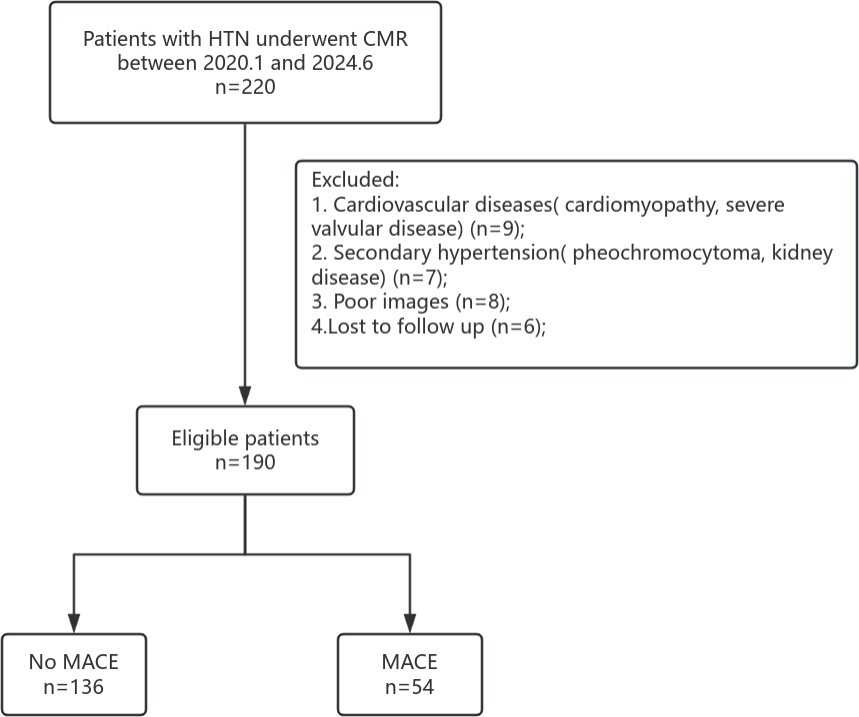
**Study flowchart for the HTN patients**. HTN, hypertension; CMR, cardiac magnetic resonance; MACE, major adverse cardiac event.

The inclusion criteria were: (1) patients met the diagnostic criteria for HTN with high cardiovascular risk; (2) patients completed a CMR examination after admission. The diagnostic criteria for HTN were based on a systolic blood pressure ≥140 mmHg and/or a diastolic blood pressure ≥90 mmHg on at least two separate office visits, or the use of medication for blood pressure control. High cardiovascular risk was diagnosed according to the HTN guidelines [2, 11, 12] of established cardiovascular disease, or the presence of at least two cardiovascular risk factors (>60 years for men or >65 years for women, type 2 diabetes, current smoker, and dyslipidemia). Comorbidities including diabetes, coronary atherosclerotic disease (CAD), myocardial infarction, heart failure (HF), and peripheral arterial disease were also assessed [11, 12]. The exclusion criteria were: (1) secondary HTN (renal artery stenosis, primary aldosteronism, polycystic kidneys, etc.); (2) cardiomyopathy (hypertrophic, dilated, and severe valvular heart disease); (3) acute myocardial infarction and HF; (4) poor image quality; (5) lost to follow-up. The final study cohort included 190 patients with HTN and high cardiovascular risk (Fig. 1). Patients were then categorized into MACE (n = 54) and non-MACE (n = 136) groups according to the follow-up results.

Laboratory test results were obtained from the electronic medical records and included N-terminal pro-brain natriuretic peptide (NT-pro-BNP), creatine kinase isoenzyme MB (CK-MB), low-density lipoprotein cholesterol (LDL-C), high-density lipoprotein cholesterol (HDL-C), triglyceride (TG), lipoprotein (a) (Lp (a)), and serum creatinine (Scr).

Patient follow-up was conducted every three months using telephone and electronic medical records by two experienced cardiologists who were blinded to the results of the CMR examination. The follow-up endpoint was the development of a MACE after discharge from hospital. This was defined as: (1) hospitalization for the occurrence of HF; (2) acute coronary syndrome (ACS); (3) stroke; (4) all-cause death [13]. According to guidelines, HF was defined by symptoms of cardiac decompensation necessitating hospital readmission, along with elevated brain natriuretic peptides and supportive evidence from cardiac imaging [14]. ACS was defined as either acute myocardial infarction, with or without ST-segment elevation, or unstable angina pectoris. Stroke was defined as an acute focal (or global) disturbance of neurological function, including ischemic stroke and hemorrhagic stroke. All-cause death was defined as death across all causes [13]. Follow-up for MACE was until September 1, 2024, with a median follow-up period of 12.0 months (interquartile range, IQR: 8.0–27.0 months).

### 2.2 CMR Acquisition

All participants underwent a CMR examination (Achieva 3.0T, Philips Medical System, Best, The Netherlands) with electrocardiographic gating and breath holds. Cine images were acquired using steady-state free-precession sequences, including short- and long-axis (2-, 3-, 4-chamber) views. Typical imaging parameters were as follows: slice thickness = 8 mm; voxel resolution = 1.8 × 1.4 × 8.0 mm^3^; repetition time = 3.1 ms; echo time = 1.54 ms; flip angle = 45°; and field of view = 350 × 350 mm^2^. LGE images were acquired 15–20 min after automated intravenous injection of 0.2 mmol/kg gadobutrol (Gd-HP-DO3A, Berlin, Germany), using the sensitive inversion recovery gradient-echo imaging sequence. Continuous slices of LGE images, including 2- and 4-chamber, as well as short-axis views, were acquired from the left ventricle apex to the base. Typical imaging parameters were as follows: voxel resolution = 1.8 × 1.4 × 8.0 mm^3^; repetition time = 5.0 ms; echo time = 2.4 ms; flip angle = 25°; and field of view = 320 × 320 mm^2^.

### 2.3 CMR Data Analysis

**Fig. 2.**
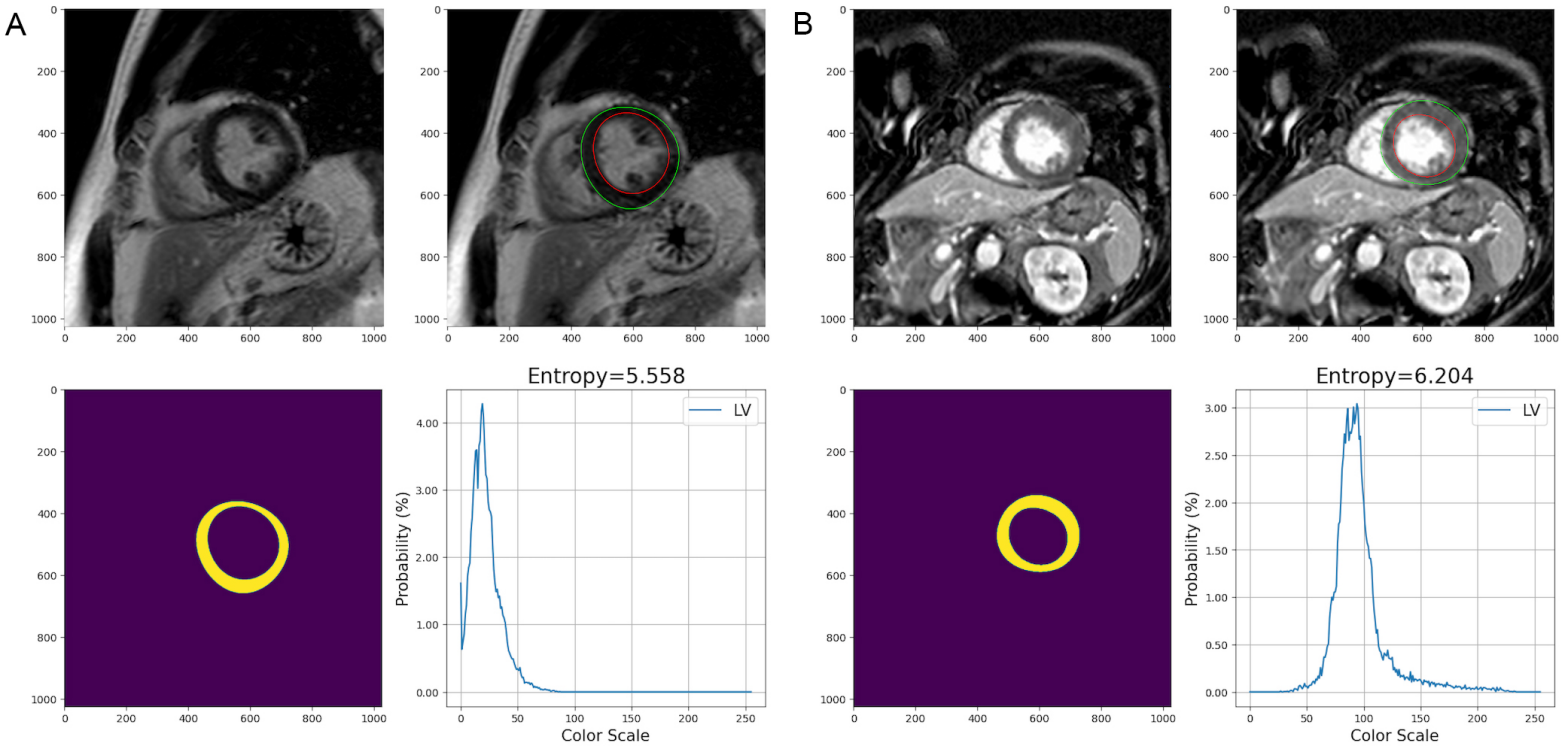
**LV entropy derived from LGE in two patients with HTN**. (A) A 49-year-old man presented with LGE in the non-MACE group during a 28-month follow-up period with LV entropy of 5.558. (B) A 53-year-old man without LGE was diagnosed with acute coronary syndrome during a 13-month follow-up period with LV entropy of 6.204. LV, left ventricular; LGE, late gadolinium enhancement.

All CMR images were post-processed using CVI42 (Circle Cardiovascular Imaging, Calgary, Alberta, Canada). Both the epicardium and endocardium were manually outlined in the short- and long-axis views. The post-processing software yields measurements for myocardial mass (MM), ventricular end-systolic volume (ESV), left ventricular end-diastolic volume (EDV), left ventricular ejection fraction (LVEF), and global longitudinal strain (GLS). Measurements for MM, EDV, and ESV were quantified relative to body surface area (BSA). LGE was defined as visible focal myocardial enhancement. The tissue characteristics of the left ventricle were measured by entropy based on the signal intensity (SI). For *P*(*x*) to be quantizable, we defined 0 to 255 to represent the SI value of each pixel point, and the frequency of each SI value was then computed to acquire *P*(*x*). The formula for entropy was as follows:

$$\mathrm{LV}\,\text{ entropy }=\sum_{x}P\,\left(x\right)\,\log P\,\left(x\right)$$

where *x* represents a specific region for each pixel point, and *P*(*x*) represents the possible dispersion of SI in this region. Examples of the derivation of LV entropy are shown in Fig. 2. An entropy value of 0 implies the region is homogeneous, while an entropy value of 10 means the region is strongly heterogeneous.

### 2.4 Reproducibility of LV Entropy

Thirty patients with HTN were selected at random to study the inter-observer reproducibility of LV entropy. The analysis was conducted by two radiologists with more than three years of diagnostic experience and who were blinded to the clinical diagnosis. Intra-observer reproducibility was also assessed in the same 30 patients by one radiologist within one month of the initial CMR examination.

### 2.5 Statistical Analysis

SPSS (version 26.0; IBM, Armonk, NY, USA) software and GraphPad Prism (version 9.0, Dotmatics, Boston, MA, USA) graphing software were used to analyze the research data. Continuous variables were expressed as the mean ± standard deviation or the median (interquartile range), and tested by one-way analysis of variance (ANOVA) or Wilcoxon rank-sum test depending on the normality of distribution. Categorical variables were expressed as frequencies with percentages and compared using the chi-square test. Variables showing significance and clinical baseline characteristics were included in univariable logistic analysis, and those showing significance in univariable logistic analysis were then included in multivariable logistic analysis. The area under the curve (AUC) derived from receiver operator characteristic (ROC) curve analysis was used to determine the diagnostic value of CMR parameters for MACEs in HTN patients. The Delong test was performed to compare the AUC for different ROC curves. Inter- and intra-observer reproducibility were evaluated using the intraclass correlation coefficient (ICC). A two-tailed *p*-value of <0.05 was considered statistically significant.

## 3. Results

### 3.1 Baseline Characteristics of HTN Patients

The baseline clinical characteristics of patients with HTN are summarized in Table 1. After excluding 30 patients who did not meet the inclusion criteria, 190 patients with high cardiovascular risk and HTN were recruited. MACEs occurred in 54 (28.4%) patients during the 12.0 month (IQR: 8.0–27.0 months) median follow-up period. These were: HF, n = 37; ACS, n = 10; stroke, n = 6; death, n = 1. The study cohort contained 128 (67.4%) males, and the mean age at presentation was 55.26 ± 12.89 years. The prevalence of HF and of current smokers was significantly higher in patients with MACE (each *p* < 0.05). NT-pro-BNP) was significantly higher in patients with MACE than in non-MACE patients (*p* < 0.05). No other significant differences were observed between the two groups.

**Table 1.** **Baseline characteristics of HTN patients**.

		Overall	MACE	Non-MACE	*p*
		(n = 190)	(n = 54)	(n = 136)	
Male, n (%)		128 (67.4)	40 (74.1)	88 (64.7)	0.214
Age, years		55.26 ± 12.89	56.85 ± 12.32	54.63 ± 13.09	0.285
BMI, kg/m^2^		25.79 ± 4.00	25.39 ± 3.61	25.94 ± 4.16	0.197
BSA, m^2^		1.77 ± 0.21	1.77 ± 0.18	1.76 ± 0.22	0.844
SBP, mmHg		136.49 ± 21.00	133.67 ± 19.38	137.61 ± 21.58	0.244
DBP, mmHg		85.68 ± 14.27	83.74 ± 14.45	86.45 ± 14.17	0.239
Duration of HTN, months		48.00 (12.00, 120.00)	54.00 (12.00, 120.00)	48.00 (12.00, 120.00)	0.615
HR, bpm		79.22 ± 27.54	80.74 ± 10.23	78.61 ± 12.02	0.253
Diabetes, n (%)		40 (21.1)	11 (20.4)	29 (21.3)	0.884
CAD, n (%)		81 (42.6)	27 (50)	54 (39.7)	0.196
MI, n (%)		18 (9.5)	7 (13.0)	11 (8.1)	0.301
HF, n (%)		51 (26.8)	25 (46.3)	26 (19.1)	<0.001*
Stroke, n (%)		52 (27.4)	17 (31.5)	35 (25.7)	0.423
Peripheral Arterial Disease, n (%)		89 (46.8)	31 (57.4)	58 (42.6)	0.066
Smoker, n (%)		65 (34.2)	25 (46.3)	40 (29.4)	0.027*
NT-pro-BNP >125 pg/mL		60 (31.6)	31 (57.4)	29 (21.3)	<0.001*
CK-MB, U/L		11.00 (9.00, 15.00)	11.50 (9.00, 17.00)	11.00 (8.00, 14.75)	0.333
LDL-C, mmol/L		2.76 ± 0.84	2.84 ± 0.84	2.73 ± 0.83	0.417
HDL-C, mmol/L		1.12 ± 0.27	1.11 ± 0.34	1.12 ± 0.24	0.881
TG, mmol/L		1.60 (1.10, 2.17)	1.67 (1.18, 2.49)	1.57 (1.08, 2.14)	0.476
Lp (a), mg/dL		7.58 (5.08, 15.75)	7.90 (5.28, 13.80)	7.55 (5.00, 17.03)	0.872
Scr, µmol/L		78.00 (68.00, 91.00)	84.00 (70.50, 95.00)	75.00 (67.00, 90.75)	0.079
Medication, n (%)					
	ACE inhibitor/ARB	70 (36.8)	19 (35.2)	51 (37.5)	0.765
	Beta-blocker	35 (18.4)	6 (11.1)	29 (21.3)	0.101
	CCB	88 (46.3)	25 (46.3)	63 (46.3)	0.997

Values are presented as the mean ± SD, median (P25, P75), numbers (percentages). BMI, body mass index; BSA, body surface area; SBP, systolic blood pressure; DBP, diastolic blood pressure; HR, heart rate; CAD, coronary atherosclerotic disease; MI, myocardial infarction; HF, heart failure; NT-pro-BNP, N-terminal pro-brain natriuretic; CK-MB, creatine kinase isoenzyme MB; LDL-C, low-density lipoprotein cholesterol; HDL-C, high-density lipoprotein cholesterol; TG, triglyceride; Lp (a), lipoprotein (a); Scr, serum creatinine; ACE, angiotensin-converting enzyme; ARB, angiotensin II receptor blocker; CCB, calcium channel blocker. * *p* < 0.05.

### 3.2 CMR Data for HTN Patients

CMR parameters are presented in Table 2. Compared with the non-MACE group, HTN patients with MACE showed significantly lower GLS and LVEF, and significantly higher LV end systolic volume index (LVESVi), LV end diastolic volume index (LVEDVi) and myocardial mass index (all *p* < 0.05). Furthermore, HTN patients with MACE had higher LV entropy and a higher prevalence of LGE compared to the non-MACE group (all *p* < 0.05). However, overlap in LV entropy values between the MACE and non-MACE groups was observed. No significant difference in maximal ventricular thickness (MVT) was observed between the two groups.

**Table 2.** **CMR data for HTN patients**.

		Overall	MACE	Non-MACE	*p*
		(n = 190)	(n = 54)	(n = 136)	
CMR indices					
	GLS, %	–11.50 ± 4.79	–8.23 ± 5.33	–12.80 ± 3.86	<0.001*
	MVT, mm	12.67 ± 3.65	12.96 ± 3.79	12.56 ± 3.60	0.501
	LVEF, %	54.63 ± 14.83	44.36 ± 19.32	58.71 ± 10.12	<0.001*
	LVESVi, mL/m^2^	39.71 ± 33.50	57.28 ± 43.95	32.73 ± 25.30	<0.001*
	LVEDVi, mL/m^2^	80.36 ± 35.85	95.03 ± 47.17	74.54 ± 28.38	0.004*
	Myocardial mass index, g/m^2^	70.41 ± 27.54	84.34 ± 32.56	64.88 ± 23.18	<0.001*
	Presence of LGE, %	64 (33.7)	25 (46.3)	39 (28.7)	0.020*
LV Entropy		5.30 ± 1.19	5.75 ± 0.89	5.12 ± 1.26	<0.001*

GLS, global longitudinal strain; MVT, maximal ventricular thickness; LVEF, left ventricular ejection fraction; LV, left ventricular; LVESVi, LV end systolic volume index; LVEDVi, LV end diastolic volume index. * *p* < 0.05.

### 3.3 Logistic Regression Analysis and Predictors of MACE in HTN

The results of logistic regression analysis are shown in Table 3. Parameters that were significantly different between the two groups of HTN patients were subjected to univariate logistic regression analysis, and those found to be significant were then included in multivariate regression analysis. Significant risk factors for the development of MACE were HF, current smoker, NT-pro-BNP, GLS, LVEF, LVESVi, LVEDVi, myocardial mass index, presence of LGE, and LV entropy (all *p* < 0.05). Additionally, subgroup analysis showed that LV entropy was a univariate predictive factor for HF (odds ratio (OR): 2.330; 95% CI: 1.404–3.868; *p* < 0.05). No significant associations were found between LV entropy and ACS, stroke, or all-cause death (all *p* > 0.05) (**Supplementary Table 1**). Independent predictors of MACE in patients with HTN were current smoking, NT-pro-BNP, GLS, LVEF, and LV entropy (all *p* < 0.05).

**Table 3.** **Univariable and multivariable logistic analysis of MACE in HTN**.

	Univariable		Multivariable	
	OR (95% CI)	*p*	OR (95% CI)	*p*
Age	1.014 (0.989–1.040)	0.284		
Male	0.642 (0.318–1.296)	0.216		
Diabetes	0.944 (0.433–2.057)	0.884		
CAD	1.519 (0.805–2.864)	0.197		
MI	1.692 (0.619–4.625)	0.305		
HF	3.647 (1.839–7.233)	<0.001*		
Stroke	1.326 (0.664–2.646)	0.424		
Peripheral Arterial Disease	1.813 (0.958–3.429)	0.067		
Smoker	2.069 (1.080–3.962)	0.028*	2.787 (1.079–7.204)	0.034*
NT-pro-BNP >125 pg/mL	4.973 (2.525–9.794)	<0.001*	2.498 (1.023–6.103)	0.045*
GLS	1.272 (1.161–1.393)	<0.001*	1.175 (1.031–1.341)	0.016*
LVEF	0.935 (0.912–0.958)	<0.001*	0.945 (0.900–0.993)	0.024*
LVESVi	1.022 (1.011–1.033)	<0.001*		
LVEDVi	1.015 (1.006–1.025)	0.001*		
Myocardial mass index	1.026 (1.013–1.039)	<0.001*		
Presence of LGE	2.144 (1.118–4.113)	0.022*		
LV Entropy	1.775 (1.236–2.549)	0.002*	1.569 (1.039–2.369)	0.032*

OR, odds ratio; CAD, coronary atherosclerotic disease; MI, myocardial infarction; HF, heart failure. *p < 0.05.

**Fig. 3.**
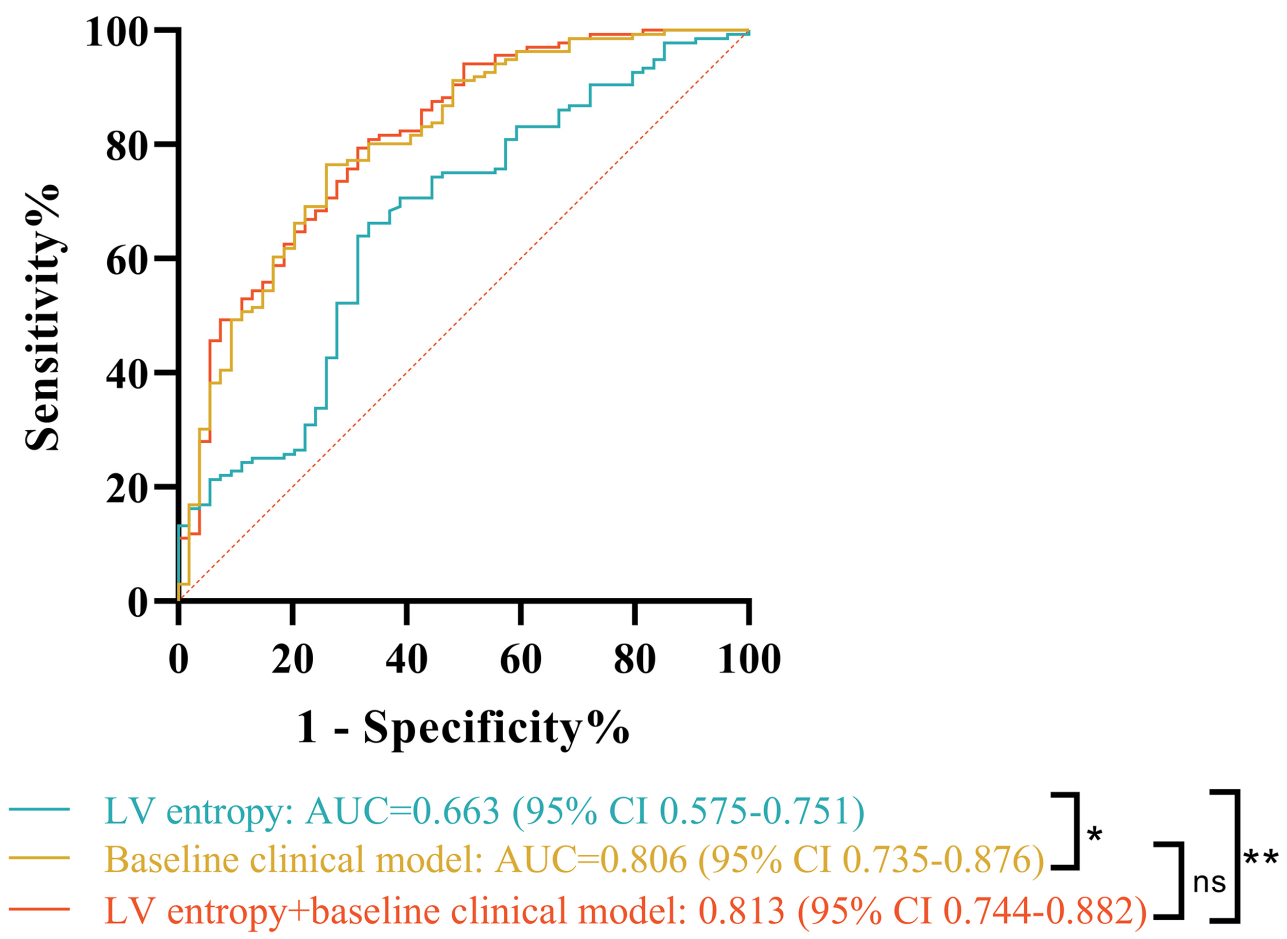
**ROC curves for prediction of adverse events**. ROC analysis of LV entropy (*p* < 0.001), the baseline clinical model (*p* < 0.001), and LV entropy + baseline clinical model (*p* < 0.001) for the prediction of MACE in patients with HTN. ROC, receiver operating characteristic; AUC, area under the curve. ns, not significant. * *p* < 0.05. ** *p* < 0.001.

The AUC of LV entropy for the prediction of MACE in patients with HTN was 0.663 (95% CI: 0.575–0.751, *p* < 0.001) (Fig. 3). The optimal LV entropy cut-off value that gave the highest sensitivity (66.7%) and specificity (66.2%) was 5.73. A higher AUC value was observed following separate analysis of the baseline clinical model, consisting of current smoking, NT-pro-BNP, GLS, and LVEF [0.806 (95% CI: 0.735–0.876)]. The addition of LV entropy to the baseline clinical model increased the AUC [0.813 (95% CI: 0.744–0.882)], although this was not significantly different to the clinical model alone (*p* = 0.57) (Table 4).

**Table 4.** **Differences in AUC between predictive models**.

	Difference in AUC	Z	*p*
Clinical model vs. Clinical model + LV Entropy	0.008	0.568	0.570
Clinical model vs. LV Entropy	0.143	2.790	0.005*
Clinical model + LV Entropy vs. LV Entropy	0.150	3.543	<0.001*

*p < 0.05

### 3.4 Intra-observer and Inter-observer Reproducibility of LV Entropy

Good reproducibility was observed for LV entropy, including for intra- (ICC 0.885, 95% CI: 0.772–0.943) and inter-observer variability (ICC 0.901, 95% CI: 0.489–0.967). Details of the ICC values are shown in **Supplementary Table 2**.

## 4. Discussion

This study investigated the predictive value of LV entropy derived from routine LGE images for MACE in patients with high cardiovascular risk and HTN over a median period of 12.0 (8.0–27.0) months follow-up. The key findings were firstly that LV entropy was significantly higher in HTN patients with MACE than in those without. Secondly, multivariate regression analysis identified LV entropy as an independent predictor for MACE in HTN patients.

Despite the numerous methods used to assess MACE, practical evaluation of poor outcomes in patients with HTN is unsatisfactory, mainly due to the significant myocardial heterogeneity in ventricular remodeling. LV entropy is a reliable parameter of image complexity that can effectively, directly, and accurately assess myocardial heterogeneity. Several studies have shown that increased LV entropy was significantly associated with worse prognosis in patients with cardiac disease. Antiochos *et al*. [9] reported that LV entropy derived from LGE was an independent predictor of MACE in patients with ventricular arrhythmias. Gu *et al*. [15] found that LV entropy was independently associated with sudden cardiac death, and could help with risk stratification of patients with hypertrophic cardiomyopathy. A recent study reported that LV entropy is a reliable predictor of MACE in patients with left ventricular noncompaction, incremental to LGE [16]. Moreover, previous studies reported higher LV entropy at presentation in coronary atherosclerotic heart disease patients with MACE, and that entropy of the peri-infarct region could improve risk stratification in patients with post-myocardial infarction [17, 18].

Building upon previous research, the present work has expanded knowledge of the prognostic value of LGE-derived LV entropy to patients with high cardiovascular risk and HTN. LV entropy in the MACE group was found to be significantly higher than in the non-MACE group, suggesting the presence of myocardial heterogeneity in the former group of patients. Myocardial heterogeneity depends on complex interactions between plasma humoral factors and ventricular wall stress [19]. Patients with MACEs presented with a higher incidence of current smoking. Cigarette smoking may predispose HTN patients to a higher risk of myocardial heterogeneity, which is affected by activation of the sympathetic nervous system, oxidative stress, and inflammation [20]. Furthermore, a recent study reported that GLS was associated with MACE in patients with HTN [21], consistent with findings from the present work. Our study also found that GLS was significantly decreased in patients with HTN, with the MACE group showing a greater decrease than the non-MACE group. Dini *et al*. [22] previously reported that reduced GLS was associated with increased LV filling pressure. Additionally, patients with MACE presented with increased LVESVi, LVEDVi, and myocardial mass index. Moreover, a previous study reported that mechanical loading and stretching of the myocardium could be initiating factors for myocardial heterogeneity [23]. Therefore, multiple pathophysiological mechanisms that produce myocardial heterogeneity may be present in hypertensive patients, and clinicians should pay more attention to changes in LV entropy.

Myocardial fibrosis leading to impaired ventricular function and deteriorated ventricular morphology is a well-recognized mechanism for MACE in patients with cardiac diseases [24]. In the present study, 25 (46.3%) of the patients with MACE presented with LGE. Furthermore, LGE was identified as a risk factor for MACE in univariate regression analysis, consistent with the results of a previous study [6]. However, LGE was not a statistically significant factor when analyzed using multivariate regression analysis, possibly due to differences in study populations. An earlier study reported that patients with HTN may present with at least two types of myocardial fibrosis, namely reactive fibrosis (or diffuse myocardial fibrosis) and reparative fibrosis (or replacement fibrosis) [10]. LGE is a non-invasive imaging modality that is commonly used to assess replacement fibrosis. However, it has limitations for the assessment of diffuse myocardial fibrosis and overall myocardial heterogeneity due to the lack of significant signal intensity thresholds. Our study also found that 29 (53.7%) patients with MACE lacked LGE. Wang *et al*. [17] found that 23.9% of CAD patients without LGE presented with MACE. Furthermore, LV entropy was an independent predictive factor for the incidence of MACE in CAD patients with and without LGE, suggesting that LV entropy can effectively demonstrate heterogeneity of the entire myocardium [16]. Gu *et al*. [15] reported that assessment of LV entropy could predict the risk of HF in patients with HCM by identifying different types of fibrosis that were indistinguishable from LGE. In the current study, we also found that LV entropy was associated with the incidence of HF (OR: 2.330; 95% CI: 1.404–3.868; *p* < 0.05), and that LGE was ineffective at predicting the occurrence of MACE. Hence, we speculate that LV entropy measurements without signal intensity thresholds can be used to assess the non-homogeneous fibrosis region. Therefore, LV entropy, as a parameter derived from LGE, could fill a gap in the assessment of CMR in the overall myocardium by identifying the presence of heterogeneous alterations in patients with HTN.

LGE-derived entropy is a novel and non-invasive parameter that can evaluate the degree of myocardial heterogeneity in patients with cardiac diseases by quantifying each myocardial pixel point. Increased entropy indicates the presence of a mixture of different types of tissues. Moreover, LV entropy derived from LGE is easily and rapidly acquired with minimal postprocessing, and covers the entire LV myocardium. In this study, the border delineation and entropy analysis for each subject took only 2 to 3 minutes. We found that LV entropy was an independent predictor for the development of MACE in HTN patients. A cut-off value of >5.73 for increased entropy showed the highest sensitivity (66.7%) and specificity (66.2%) for identifying HTN patients with MACE. A previous study reported that T1 mapping and extracellular volume (ECV) could effectively capture diffuse myocardial fibrosis. However, ECV is dependent on the accuracy of T1 mapping and the hematocrit, and abnormal ECV needs to be used in conjunction with LGE [25, 26]. LV entropy derived from LGE can easily quantify every myocardial pixel point to demonstrate overall myocardial heterogeneity. However, although LV entropy exhibited a significant prognostic value in MACE prediction in this study, its clinical application may be limited by the overlap in LV entropy values between patients with and without MACE. The considerable overlap in LV entropy measurements obtained from CMR for predicting MACE strongly suggests that clinical management should not rely on LV entropy alone. Interpreting LV entropy alongside other parameters is essential for accurate risk stratification in patients with HTN.

## 5. Limitations

Several limitations of this study should be acknowledged. First, it was a single-center retrospective study with small sample size, and all patients were examined on the same MRI instrument for CMR. The relatively small sample size reduces the statistical power, and hence application of the predictive model is limited. The generalizability of entropy values and their predictive efficacy with different MRI instruments and scanner field strengths requires further validation through multicenter, randomized controlled studies with large sample size. Second, this study did not analyze CMR T1 mapping and ECV due to missing data in some cases, and thus comparison of LV entropy with ECV was not performed. We intend to address this limitation by expanding relevant population studies and by incorporating the CMR mapping technique in future research. Third, we did not investigate entropy between HTN patient subgroups with different durations and classifications. Our group will conduct further advanced studies to explore the predictive value of entropy in different HTN populations.

## 6. Conclusions

In conclusion, patients with MACEs presented with higher LV entropy derived from LGE images. Furthermore, LV entropy has the potential to differentiate cardiac patients with different risk levels, and to assess myocardial heterogeneity in patients with high cardiovascular risk and HTN. Although LV entropy shows predictive value for adverse events, some overlap occurs between the MACE and non-MACE groups. Hence, higher LV entropy needs to be interpreted within the context of contemporary clinical data.

## Availability of Data and Materials

The raw datasets supporting the findings of this study are available from the corresponding author upon reasonable request.

## Supplementary Material

Supplementary material associated with this article can be found, in the online version, at https://doi.org/10.31083/RCM26499.
