# Supplementary material for: Increased Entropy Predicts Adverse Cardiac Events in Patients with High Cardiovascular Risk and Hypertension: A Novel Imaging Parameter Derived from Late Gadolinium Enhancement
Source: Rev Cardiovasc Med. 2025 May 27;26(5):26499. doi: 10.31083/RCM26499 (PMC12135657; doi:10.31083/RCM26499)
Supplement: Supplementary file 1 [file 2153-8174-26-5-26499-s1.docx]

**Supplementary Table 1**

Univariable association of LV entropy with HF, ACS, stroke and all cause death

|  | OR (95% CI) | P |
| --- | --- | --- |
| Hospitalization for the occurrence of HF |  |  |
| LV entropy | 2.330 (1.404-3.868) | 0.001* |
| ACS |  |  |
| LV entropy | 1.095 (0.620-1.933) | 0.754 |
| Stroke |  |  |
| LV entropy | 1.307 (0.571-2.992) | 0.526 |
| All cause death |  |  |
| LV entropy | 0.500 (0.141-1.773) | 0.284 |

LV, left ventricular; HF, heart failure; ACS, acute coronary syndromes;

* P<0.05.

**Supplementary Table 2**

Intra-observer and inter-observer reproducibility of LV entropy

|  | **Inter-observer** |  | **Intra-observer** |  |
| --- | --- | --- | --- | --- |
|  | ICC | 95% CI | ICC | 95% CI |
| LV entropy | 0.885 | 0.772-0.943 | 0.901 | 0.489-0.967 |

ICC, intraclass correlation coefficient; LV, left ventricular.
